# miRNAs associated with chemo-sensitivity in cell lines and in advanced bladder cancer

**DOI:** 10.1186/1755-8794-5-40

**Published:** 2012-09-06

**Authors:** Iver Nordentoft, Karin Birkenkamp-Demtroder, Mads Agerbæk, Dan Theodorescu, Marie Stampe Ostenfeld, Arndt Hartmann, Michael Borre, Torben F Ørntoft, Lars Dyrskjøt

**Affiliations:** 1 Department of Molecular Medicine, Aarhus University Hospital, Aarhus, Denmark; 2 Department of Oncology, Aarhus University Hospital, Aarhus, Denmark; 3 University of Colorado Comprehensive Cancer Center, Aurora, CO 80045, USA; 4 Department of Pathology, University of Erlangen, Erlangen, Germany; 5 Department of Urology, Aarhus University Hospital, Aarhus, Denmark

## Abstract

**Background:**

MicroRNA is a naturally occurring class of non-coding RNA molecules that mediate posttranscriptional gene regulation and are strongly implicated in cellular processes such as cell proliferation, carcinogenesis, cell survival and apoptosis. Consequently there is increasing focus on miRNA expression as prognostic factors for outcome and chemotherapy response. Only approximately 50% of patients with bladder cancer respond to chemotherapy. Therefore, predictive markers, such as miRNAs, that can identify subgroups of patients who will benefit from chemotherapy will have great value for treatment guidance.

**Methods:**

We profiled the expression of 671 miRNAs in formalin fixed paraffin embedded tumors from patients with advanced bladder cancer treated with cisplatin based chemotherapy. We delineated differentially expressed miRNAs in tumors from patients with complete response vs. patients with progressive disease and in tumors form patients with short and long overall survival time. Furthermore, we studied the effect of up- and down regulation of key miRNAs on the cisplatin sensitivity in eight bladder cancer cell lines with different sensitivities to cisplatin.

**Results:**

miRNA expression profiling identified 15 miRNAs that correlated with response to chemotherapy and 5 miRNAs that correlated with survival time. Three miRNAs were associated with both response and survival (886-3p, 923, 944). By changing the cellular level of the response-identified miRNAs in eight bladder cell lines with different cisplatin sensitivity we found that down-regulation of miR-27a, miR296-5p and miR-642 generally reduced the cell viability, whereas up-regulation of miR-138 and miR-886-3p reduced the viability of more than half of the cell lines. Decreasing miR-138 increased the cisplatin sensitivity in half of the cell lines and increasing miR-27a and miR-642 generally increased cisplatin sensitivity.

**Conclusions:**

MiRNAs seem to be involved in cisplatin based chemo response and may form a new target for therapy and serve as biomarkers for treatment response.

## Background

Bladder carcinoma is the fourth most common cancer in men in the Western world. The disease is characterized by frequent recurrences and poor clinical outcome when tumors progress to invasive disease. The most prevalent histopathologic type of bladder cancer in Western countries is transitional cell carcinoma (TCC) accounting for up to 95% of all cases. About 30% of patients with TCC’s either present with or develop invasion into the detrusor musculature, a prognostic indication carrying an about 50% risk of fatal outcome following development of metastatic disease dissemination. In patients with locally advanced or metastatic disease, the response rate to chemotherapy is 30-50% [1]. Presently, there are two standard chemotherapeutic regimens for advanced urothelial carcinomas: MVAC (Methotrexate, vinblastine, doxorubicin, and cisplatin) and GC (gemcitabine and cisplatin). Median survival in these patients is around 15 months, and the 5-year overall survival rate is about 15% [2]. Although the gemcitabine and cisplatin combination has a significantly better toxicity profile, both regimens still carry risk for significant toxicity and toxic deaths [3], and a substantial fraction of patients will suffer from adverse reactions without achieving clinical benefit. Early, or even pretherapeutic, discrimination between likely responders and non-responders would greatly improve selection of patients to chemotherapy and thereby benefit both groups.

Deregulation of microRNA (miRNAs or miRs) levels is associated with dysplasia and cancer, and miRNA profiles have been used to classify human cancers and predict outcome more accurately than mRNA expression profiles [4-10]. Furthermore, urinary miRNAs have been shown to be clinically useful for noninvasive bladder cancer diagnostics (miR-452 and miR-222) [9].

miRNAs are endogenous, non coding RNA molecules of approximately 19-25 nucleotides in length. Most miRNAs represses mRNA translation by blocking of translation, less frequently mRNA degradation/deadenylation, however a minor proportion of the miRNAs mediate mRNA target up-regulation [11,12]. Due to the low stringency of the required binding of 6-8 bases of the seed sequence of the miRNA to the mRNA, each miRNA can potentially interact with hundreds of mRNA targets. With the more than 1576 identified unique human miRNAs (miRBase, version 18) it is predicted that around 30% of the transcriptome is regulated by miRNAs. miRNA genes are frequently located in cancer-associated genomic regions of loss of heterozygosity, amplified regions, or fragile sites [13]. Aberrantly expressed miRNAs have been shown to be associated with many types of cancers. miRNAs can function as both oncogenes (onco-miRs) or tumor suppressors [14-16]. Therefore expression profiles of miRNAs may provide information about chemotherapy sensitivity prior to treatment and changes in miRNA expression during treatment could be a marker for chemotherapy response. Improvements in high throughput miRNA profiling have provided increasing evidence of miRNA deregulation in drug resistant and sensitive cells. Blower and colleagues investigated miRNA expression profiles in the NCI-60 cancer cell panel and showed correlation between chemotherapy potency and miRNA expression patterns [15]. Kovalchuk, Pogribny and colleagues identified 137 deregulated miRNAs when comparing doxorubicin resistant and sensitive MCF-7 breast cancer cell lines and 103 deregulated miRNAs when comparing cisplatin resistant and sensitive MCF-7 cells [17-19]. Boren and colleagues investigated 16 different ovarian cancer cell lines and found 27 (of 335 profiled) miRNAs correlated to response to one or more drugs (cisplatin, gemcitabine, docetaxel, doxorubicin, topotecan, paclitaxel) [17]. In human epithelial ovarian cancer samples Yang and colleagues found 34 out of 326 miRNAs were associated with response to platinum based chemotherapy (complete response (*n* = 42) and non-complete response groups (*n* = 27))[20].

To gain information on the potential role of miRNAs in drug response we profiled miRNA expression in bladder tumors from patients having either complete response or progressive disease after cisplatin based chemotherapy. Furthermore, we studied the effect on cisplatin sensitivity in different bladder cancer cell lines (UMUC9, UMUC14, SLT4, 253JBV, RT4, CLR2169, HT1197, 575A) by changing the levels of the miRNAs predictive of chemotherapy response. Our findings show that 15 miRNAs correlated with response to chemotherapy, and 5 miRNAs were associated with survival time. Three miRNAs were associated with both response and survival (886-3p, 923, 944). By changing the cellular level of the treatment-response associated miRNAs in eight bladder cell lines with different cisplatin sensitivity we found that down-regulation of miR-27a, miR296-5p and miR-642 reduced the cell viability, whereas up-regulation of miR-138 and miR-886-3p reduced the viability of more than half of the cell lines. Decreasing miR-138 increased the cisplatin sensitivity in half of the cell lines and increasing miR-27a and miR-642 increased cisplatin sensitivity.

## Methods

### Patient samples

The study included a total of 30 patients receiving first line platinum containing combination chemotherapy for locally advanced (T4B, N2-3) and/or metastatic (M1) transitional cell carcinoma (TCC) at the Department of Oncology, Aarhus University Hospital, Denmark, in the 10-year period between 1991 and 2001. All patients had histologically confirmed transitional cell carcinoma of the urothelial tract (urethra, bladder, ureters or renal pelvis). All patients received cisplatin-based combination chemotherapy schedules (Additional file 1: Tables S1 and Additional file 2: Table S2) and were included in either one of four phase II protocols, one phase III protocol, or were treated with the standard regimen at the relevant time (MVAC or GC). Patients were treated to a maximum of 6 cycles unless progression or unacceptable toxicity appeared. Patients receiving standard treatment were prospectively followed under the same conditions as patients in protocols [21]. Patients presenting with locally advanced disease, obtaining a significant partial or complete response to chemotherapy, were offered consolidating surgery or, more often, radiotherapy when applicable [21].

### Cell culture

Bladder cell lines UMUC9, UMUC14, SLT4, 253JBV, RT4, CLR2169, HT1197, 575A [22] (provided by Professor Dan Theodorescu, University of Colorado Comprehensive Cancer Center) were re-authenticated via STR analysis using the Cell-ID-system (G9500, Promega, Nacka, Sweden), products were analyzed using an Applied-Biosystems 3130 Genetic Analyzer. No Mycoplasma contamination was detected using nested PCR-based Mycoplasma detection. SLT4, 253JBV, RT4, CLR2169 cells were cultured in DMEM + with L-glutamine (Gibco, Invitrogen Corporation, Carlsbad, CA, USA) supplemented with 10% fetal bovine serum (Gibco), 100 U/mL penicillin (Gibco) and 100 U/mL streptomycin (Gibco). UMUC9, UMUC14, 253JBV, HT1197, 575A cells were cultured in MEM with L-glutamine (Gibco, Invitrogen Corporation, Carlsbad, CA, USA) supplemented with 1 mM Sodium Pyruvate + 0,1 mM nonessential amino, 10% fetal bovine serum (Gibco), 100 U/mL penicillin (Gibco) and 100 U/mL streptomycin (Gibco). All cells were maintained in a humidified atmosphere at 37°C and 5% CO_2_.

### Transient transfection

miRNA molecules (Ambion, Austin, TX, USA) and LNA knockdown molecules (Exiqon) were reverse Transfected using Lipofectamine 2000 (Invitrogen) according to manufacturer's guidelines. Non-targeting miRNA (Ambion, Austin, TX, USA) and non-targeting LNA molecules (Exiqon, DK) were used as controls. In short 4x10^3^ cells in 100 μL culture medium were added to wells of 96 well plates containing 50 μL miRNAs or LNA antagonism giving a final concentration of 20 nM and 40 nM respectively and then incubated at 37°C and 5% CO_2_. Cisplatin (Sigma Aldrich, Germany) was added 48 h post transfection in experiments studying the effect of chemotherapy, by first removing 50μL from each well and then adding 100μL new medium containing cisplatin at 2 times the desired concentration and then incubated for an additional 48 h at 37°C and 5% CO_2_. Finally viability was measured using the MTT assay (see section: Cell viability assay). mercury LNA™ microRNA inhibitors (EXIQON) used: hsa-miR-27a (410167-00), hsa-miR-138 (412560-00), hsa-miR-296-5p (410171-00), hsa-miR-642 (410409-00), hsa-miR-886-3p(410445-00) and mercury LNA™ microRNA inhibitors negative control A (199004-04). The miRNA molecules used for transfection are chemically modified double-stranded RNA molecules designed to mimic endogenous mature miRNA molecules: hsa-miR-27a (PM10939), hsa-miR-138 (PM11727), hsa-miR-296-5p (PM10609), hsa-miR-410 (PM11119), hsa-miR-642 (PM11477), hsa-miR-886-3p(410445-00), Pre-miR™ miRNA Precursor Molecules Negative Control #1(AM17110). Prior to miRNA transfection experiments we performed transfection with a cy3 labeled siRNA molecule to monitor successful uptake under fluorescence microscope.

### Isolation of RNA

Total RNA was isolated from formalin-fixed paraffin embedded (FFPE) tissues specimens as follows. Haematoxylin- and eosin-stained (HE) sections of the FFPE blocks were examined and areas with carcinoma cells circled and the section was photocopied onto transparent paper. Using this as a guide, an appropriate area of the block was sampled using a disposable punch biopsy needle (1.5 mm, Miltex GmbH, Germany). The sample cylinder was dispensed and minced into small pieces with a scalpel. Total RNA (range 1200 ng – 2300 ng) was then prepared using the miRNeasy® FFPE Kit (Qiagen, Germany), essentially as specified by the manufacture. The miRNeasy FFPE kit is specifically designed for FFPE material and contains reagents to reverse formalin cross linking of RNA. Furthermore the kit is developed to allow purification of all usable RNA larger than 18 nucleotides.

Cell line RNA was isolated from 6-well and 24-well plates using RNeasy spin columns (Qiagen, Hilden, Germany), as recommended by the manufacturer. All RNA samples were quantified using an Infinite® 200 PRO NanoQuant spectrophotometer (Tecan, Switzerland).

### miRNA profiling using Taqman Micro fluidic cards

Quantification of miRNAs from tissue samples was carried out by a two-step procedure using the Taqman LDA Human microRNA Panel v3.0 (Micro fluidic card, Applied Biosystems, Foster City, CA) according to the manufacturer's protocols. Taqman LDA Human microRNA Panel v3.0 was designed using Sanger miRBase v14. Briefly, 500 ng of total RNA was used for multiplexed RT reactions followed by pre-amplification. Cycling conditions were chosen according to the manufacturer's protocols. The ABI7900 HT platform with TLDA upgrade was used and data was analyzed using RQ Manager Software provided by Applied Biosystems.

### Quantitative real-time PCR

Quantification of miRNAs by Taqman real-time PCR was carried out as described by the manufacturer (Applied Biosystems). Briefly, 500 ng of template RNA was reverse transcribed by using the Taqman MicroRNA Reverse Transcription Kit and the multiplex RT primer pools containing miRNA-specific stem-loop primers (Applied Biosystems). Diluted RT product (1.5 μL) was introduced into the 20 μL PCR amplifications, which were incubated in 384-well plates on the ABI 7900HT thermo cycler (Applied Biosystems) at 95°C for 10 minutes, followed by 40 cycles of 95°C for 15 seconds and 60°C for 1 minute. Gene expression was normalized using expression of miRNA-193b. Taqman® MicroRNA Assays (Applied Biosystems, Foster City, CA) used: hsa-miR-27a (Assay ID000408), hsa-miR-138 (Assay ID002284), hsa-miR-193a-5p (Assay ID 002281), hsa-miR-193b (ID 002367), hsa-miR-296-5p (Assay ID 000527), hsa-miR-410 (Assay ID001274), hsa-miR-492 (Assay ID001039), hsa-miR-639 (Assay ID001583), hsa-miR-642 (Assay ID 001592), hsa-miR-886-3p(Assay ID002194), hsa-miR-944 (Assay ID002189).

### Cell viability assay

The viability of sub-confluent cells was analyzed by 3-(4,5-dimethylthiazole-2-yl)-2,5-diphenyltetrazolium bromide (MTT) reduction assay. The assay was performed in 96 well plates with 200μL/well. 100 μL culture medium was carefully removed and 25 μL MTT solution was added (1 g MTT (Sigma M5655) dissolved in 200 mL D-PBS.) and stored shielded from light 1.5 h at 37°C and then 100μL solubilization (50% dimethylformamide, 20% SDS) buffer was added and left protected from light overnight. Readout was done using a micro plate reader (Lab systems Multiscan MCC/340) at 540 nm. Absorbance at 692 nm was used as reference.

### Ethics statement

All patients gave their written informed consent, and the study was approved by the Central Denmark Region Committees on Biomedical Research Ethics (1994/2920).

### Statistical analysis

Selection of differentially expressed miRNAs were based on *t*-test statistics (p < 0.05) and simultaneously on assessment of false discovery rates (FDR) to correct for multiple testing. We used Significance Analysis of Microarrays (SAM) software for estimating FDR. In the analysis of treatment response we used a maximum of 10% in FDR as cutoff and in the analysis of survival we used a maximum of 50% in FDR as cutoff. FDRs for the individual miRNAs are reported.

## Results

### miRNAs differentially expressed between chemotherapy responders and non-responders

**Figure 1:**
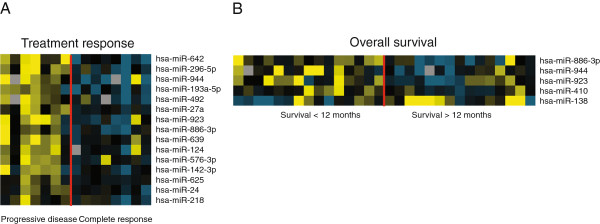
**Heatmaps of miRNA expression patterns.** miRNA expression is depicted according to (**A**) treatment response and (**B**) overall survival. Yellow; upregulation of the gene, blue; downregulation, black; median expression, grey; missing value.

We profiled the expression of 671 miRNAs in tumors from patients with advanced bladder cancer subsequently treated with cisplatin based chemotherapy (Table 1 and Additional file 1: Table S1). Seven patients experienced progression of disease (PD) and eight patients experienced complete responses (CR) according to the RECIST criteria [23]. Differentially expressed miRNAs (PD vs.CR) are listed in Table 2. The expression levels of the 15 top ranked miRNAs in the different tumors samples are shown in Figure 1A. Data was normalized using miRNA-193b as this was identified as the most stably expressed miRNA across all samples by Norm Finder [24]. Different normalization approaches showed highly similar results (Additional file 3: Table S3). As a technical evaluation of the Taqman Human Array MicroRNA Cards we selected the 5 top ranked miRNAs from the analysis (miRNA-642, 492, 27a, 296-5p, 944, 193a-5p) and performed single-plex qRT-PCR. The qRT-PCR fully confirmed the Taqman Human Array MicroRNA results (Additional file 4: Table S4). Interestingly, the analysis showed that the majority of top ranked miRNAs had a reduced expression in the CR group compared to the PD group. The chromosome location of the 15 differentially expressed miRNAs (Table 2) shows that the unidirectional expression pattern is not due to regional epigenetically silencing of the miRNAs.

**Table 1:** Patient characteristics and clinical parameters

	**Response cohort**		**Survival cohort**	
	**PD**	**CR**	**SS**	**LS**
**RECIST Category**				
CR		8	1	7
PR			2	5
PD	7		7	1
NC			3	0
NE			2	2
**In total**	7	8	15	15
**Age**	57.7 (45-62)	56.3 (38-69)	59.3 (37-75)	58.9 (38-74)
**Sex**				
Female	2	2	4	5
Male	5	6	11	10
**Overall survival**	4.7 (0.13-13.3)	39.8 (13.3-92.1)	5.3 (0.13-10.9)	59.7 (13.2-125.1)

*CR*: complete response; *PR*: partial response; *PD*: progression of disease; *NC*: no change; *NE*: not estimated; *SS*: short survival, *LS*: long survival.

**Table 2:** miRNAs differentially expressed between patients with progressive disease and complete response

**miRNA**	**Chromosome band**	**Fold change (PD vs. CR), log2**	p-value*	**FDR**
hsa-miR-642	19q13.32	-3.8	0.001	0.00
hsa-miR-296-5p	20q13.32	-2.8	0.003	0.00
hsa-miR-944	3q28	-3.6	0.006	0.00
hsa-miR-193a-5p	17q11.2	-2.6	0.009	0.00
hsa-miR-492	12q22	-2.9	0.003	0.00
hsa-miR-27a	19:p13.13	-1.8	0.003	0.00
hsa-miR-923	28S rRNA fragment	-2.5	0.021	0.00
hsa-miR-886-3p	5q31.1	-2.1	0.006	0.00
hsa-miR-639	19p13.12	-1.8	0.023	0.00
hsa-miR-124	8p23.1	-1.8	0.040	6.90
hsa-miR-576-3p	4q25	-1.6	0.025	6.90
hsa-miR-142-3p	17q22	-1.8	0.050	9.20
hsa-miR-625	14q23.3	-1.2	0.022	9.20
hsa-miR-24	9q22.32	-1.3	0.022	9.20
hsa-miR-218	4p15.31	-1.5	0.042	9.20

*PD*: progression of disease, *CR*: complete response, *FDR*: false discovery rate.

*t-test statistics.

### miRNAs differentiating short and long overall survival time

We also delineated miRNAs that correlated with survival time. We compared tumors from patients with short survival (SS, 15 patients, overall survival < 1 year) to tumors from patients with long survival (LS, 15 patients, overall survival 1 > year) patient survival cohort. Patient survival cohort contained the 15 patients from response cohort plus 15 additional patients (Table 1, Additional file 2: Table S2). Differentially expressed miRNAs (SS vs.LS) are listed in Table 3 and expression levels are shown in Figure 1B. Again Norm Finder was used to calculate the best normalize miRNA from the dataset (miRNA-324-3p). Other normalization approaches were applied as before with highly similar results (Additional file 5: Table S5). As expected we found an overlap of differentially expressed miRNAs between the analyses performed for patient response cohort and patient survival cohort (miRNA-886-3p, miRNA-923 and miRNA-944).

**Table 3:** miRNAs differentially expressed between patients with short and long survival

**miRNA**	**Chromosome band**	**Fold change (SS vs. LS), log2**	p-value*	**FDR**
hsa-miR-886-3p	5q31.1	-1.6	0.011	0.00
hsa-miR-944	3q28	-1.8	0.018	0.00
hsa-miR-923	28S rRNA fragment	-1.5	0.018	0.00
hsa-miR-410	14q32.31	-1.15	0.011	25.45
hsa-miR-138	3p21.32	1.94	0.022	42.42

*SS*: short survival, *LS*: long survival, *FDR*: false discovery rate.

*t-test statistics.

### Cisplatin sensitivity of bladder cell lines

**Figure 2:**
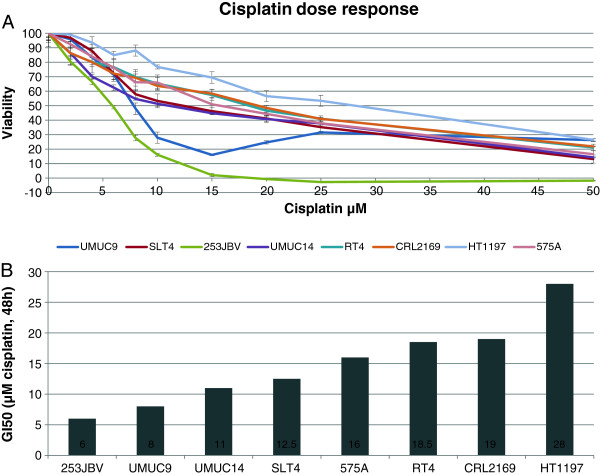
**Single agent dose-dependent cytotoxicity induced by cisplatin.** Human bladder cancer cells UMUC9, UMUC14, SLT4, 253JBV, RT4, CLR2169, HT1197, 575A were seeded in 96 well plates and treatment with the indicated cisplatin concentration started 48 h after seeding and continued for 48 h. Cell viability was assessed with MTT-assay. Panel A: cell viability cisplatin dose response curve. Panel B: Cisplatin concentration killing 50% of the cells after 48 h relative to non-treated cells (GI50, Growth inhibition 50%). GI50 was estimated based on two independent experiments. Each drug concentration was tested in six individual wells.

**Figure 3:**
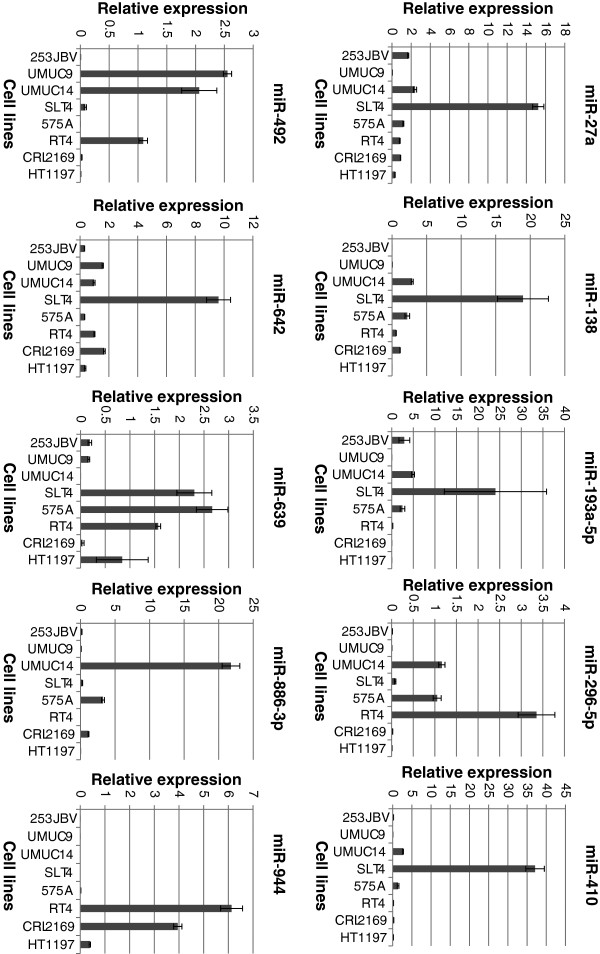
**Expression of mature miRNA in bladder cell lines.** Expression of mature miR-27a, miR-138, miR-193a-5p, miR-296-5p, miR-410, miR-492, miR-642, miR-886-3p, miR-923 and miR-944 in bladder cell lines 253JBV, UMUC9, UMUC14, SLT4, 575A, RT4, CLR2169 and HT1197 were determined using real-time Q-PCR (n = 3). miR-193b expression was used as normalizer. Cell lines are listed according to Cisplatin GI50 sensitivity.

In order to investigate the ability of the differentially expressed miRNAs from patient response cohort to predict the sensitivity to cisplatin based chemotherapy we exposed a range of bladder cancer cell lines (UMUC9, UMUC14, SLT4, 253JBV, RT4, CLR2169, HT1197, 575A) to cisplatin. These cell lines have previously been documented to exhibit differing sensitivity to cisplatin. Above the cell are listed from high to low cisplatin sensitivity. The concentration of cisplatin required to inhibit the cell growth by 50% (GI50) during 48 h incubation in our laboratory was calculated from dose response curves (Figure 2). The studies were conducted with equal cell seeding density to avoid any confluences-related difference in effect observed on cell viability. Next we measured the expression of miRNA-27a, miRNA-138, miRNA-193a-5p, miRNA-296-5p, miRNA-410, miRNA-492, miRNA-642, miRNA-886-3p, miRNA-923 and miRNA-944 using qRT-PCR in the 8 cell lines (Figure 3). We observed no association between cisplatin GI50 and miRNA expression in the cell lines.

### Cisplatin sensitivity following miRNA knock down

**Figure 4:**
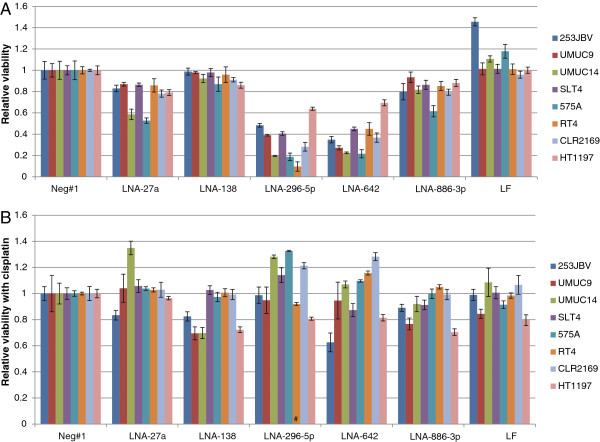
**miRNA down-regulation in bladder cell lines.** LNA based antagomirs were used to silence miR-27a, miR-138, miR-296-5p, miR-642 and miR-886-3p in the bladder cell lines 253JBV, UMUC9, UMUC14, SLT4, 575A, RT4, CLR2169 and HT1197. LNA knockdown molecules were reverse transfected at 20nM (n = 8), and after 48 h incubation culture media, with or without cisplatin (GI50), was added to the cells (n = 4). The viability of the cells was determined after 48 h incubation (96 h post transfection) by MTT-assay. LF2000 designate controls using the transfection reagent alone. **A**: Relative cell viability expressed as the viability of the antagomiR transfected cells normalized to the negative control molecule for each cell line. **B**: Relative cell viability expressed as the viability of the antagomiR transfected cells treated with cisplatin (GI50) compared to the antagomiR transfected cells without cisplatin normalized to the negative control molecule for each cell line. (#: the high reduction of cell viability imposed by the antagomirs prohibits precise measure of effect of cisplatin treatment during miR down regulation).

To further investigate the role of the identified miRNAs in treatment response and survival time of cancer patients, we knocked down five of these key miRNAs (miRNA-27a, miRNA-138, miRNA-296-5p, miRNA-642, miRNA-886-3p) using LNA-anti-miRs. 48 hours after transfection the cells were incubated with cisplatin (GI50) for another 48 hours followed by viability analysis. The experiments were conducted in all eight bladder cancer cell lines to avoid any single cell line-dependent phenomenon and to enable drawing of more general conclusions. miRNA down regulation was verified by qRT-PCR following knockdown using two LNA-anti-miRs (Additional file 6: Figure S1). First, the effect of miRNA knock down was measured using MTT assays (Figure 4A). The viability of the cell lines *per se* was clearly reduced following down-regulation of miR-296-5p and miR-642, and reduced to some extent following down-regulation of miR-27a and miR-886-3p. Next, the effect of miRNA-knock down on cisplatin sensitivity was measured by incubating Transfected cells with the estimated GI50 concentration of cisplatin. Cisplatin was added 48 h post transfection and incubated for additional 48 h followed by MTT viability measurement (Figure 4B). This revealed no clear correlation between cisplatin sensitivity and miRNA knock down, however down-regulation of miR-138 increased the cisplatin sensitivity of four of the eight tested cell lines by approximately 20% (Figure 4B). The cellular response in cisplatin treated cells to miR-296-5p and miR-642 transfection was more difficult to interpret as these miRNAs already showed reduced viability following the miRNA transfection.

### Cisplatin sensitivity following miRNA knock in

**Figure 5:**
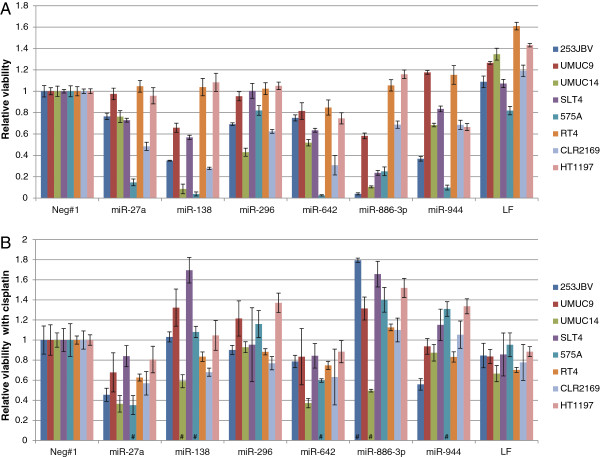
**miRNA up-regulation in bladder cell lines.** miRNA molecules were used to up-regulate the levels of miR-27a, miR-138, miR-296-5p, miR-642, miR-886-3p and miR-944 in the bladder cell lines UMUC9, UMUC14, SLT4, 253JBV, RT4, CLR2169, HT1197 and 575A. miRNAs molecules were reverse transfected at 40 nM (n = 8) and after 48 h incubation culture media with or without cisplatin (GI50) added to the cells (n = 4). The viability of the cells was determined after 48 h incubation (96 h post transfection) by MTT-assay. LF2000 designate controls using the transfection reagent alone. **A**: Relative cell viability expressed as the viability of the miRNA transfected cells normalized to the negative control molecule for each cell line. **B**: Relative cell viability expressed as the viability of the antagomiR transfected cells treated with cisplatin (GI50), compared to the antagomiR transfected cells without cisplatin, normalized to the negative control molecule for each cell line. (#: the high reduction of cell viability imposed by the precursor miRs prohibits precise measure of effect of cisplatin treatment during miR down regulation).

We also analyzed the effect of miRNA overexpression using synthetic precursor miRNAs (miRNA-27a, miRNA-138, miRNA-296-5p, miRNA-642, miRNA-886-3p, miRNA-944). First, we measured the effect of miRNA overexpression using MTT assays (Figure 5A). Cell viability after miRNA transfection varied extensively, however especially knock in of miR-138 and miR-886-3p had a highly deleterious effect on cell viability in half of the analyzed cell lines. Next we measured the effect of miRNA knock in on cisplatin sensitivity by incubating transfected cells with the estimated GI50 concentration of cisplatin. Cisplatin was added 48 h post transfection and incubated for additional 48 h followed by MTT viability measurement (Figure 5B). This revealed that miR-27a and miR-642 overexpression increased the cisplatin sensitivity in most of the cell lines. The effect of miR-138 and miR-886-3p overexpression on cisplatin sensitivity cannot be determined due to the highly reduced viability by the miR alone.

## Discussion

Here we performed miRNA expression profiling of tumors from patients with advanced bladder cancer that showed progressive disease, or complete response, following cisplatin based chemotherapy according to the RECIST criteria [23].

The miRNA profiling identified a number of down-regulated miRNAs that were correlated with chemotherapy response. Among these were miRNA-27a (3.5 fold), miRNA-296-5p (7 fold), miRNA-642 (14 fold), miRNA-886-3p (4.3 fold). Mature miR-27a and miR-886-3p transfection reduced the viability of the tested cell lines marginally; however the cisplatin sensitivity was not increased. Knock in of miRNA-296-5p, miRNA-642 resulted in reduced viability of all eight cell lines tested but the cisplatin sensitivity was unaltered (Figure 4A3B). miR-27a is part of the miRNA cluster miR-23a ~ 27a ~ 24-2 and functions as an oncogene in gastric cell lines (MKN45, MGC803) and the tumor suppressor Prohibitin is up regulated both at the transcriptional and translational level by antimiR-27a [25,26]. miR-27a has been shown to bind directly to the mRNA coding for the p44 subunit of general transcription factor IIH (TFIIH) during G2-M transition resulting in destabilizing the TFIIH complex and modulate the transcriptional shutdown during the G2-M phase [27]. Over expression of miR-23a ~ 27a ~ 24-2 sensitized HEK293T cells to TNF-α cytotoxicity suggesting it to be of value in cancer therapy [28,29].

Among the identified miRNAs, mir-138 was the only miRNA up-regulated more than 2 fold (3.8 fold) in the survival group (Table 2). In leukemia cell line HL-60/VCR miR-138 was demonstrated to significantly down-regulate the expression of P-glycoprotein, Bcl-2, and the transcription of the multidrug resistance gene 1 (MDR1) [30]. In human osteosarcoma cell line, U2OS MiR-138 was shown to directly target the histone γH2AX 3'-UTR and reduce histone γH2AX expression, and induce chromosomal instability after DNA damage. MiR-138 over expression enhanced cellular sensitivity to cisplatin and reintroduction of histone γH2AX in miR-138 overexpressing cells attenuated the sensitization to cisplatin [31]. miR-138 is inversely correlated with excision repair cross-complementing rodent repair deficiency, complementation group 1 (*ERCC1)* expression in the A549/DDP multidrug-resistant human lung adenocarcinoma cells. Furthermore, the enforced increase of miR-138 levels in the A549/DDP cells down-regulates expression of ERCC1 and increases sensitivity of the drug-resistant cancer cells to cisplatin by inducing apoptosis [32]. Based on this we would expect increased cisplatin sensitivity when overexpressing mature miR-138. This was only observed in three of the eight tested bladder cell lines (UMUC14, RT4, CLR2169, Figure 5B), however overexpression of miR-138 in itself reduced the viability markedly of half of the cell lines (Figure 5A), and so the effect of subsequent cisplatin exposure is difficult to interpret. Down-regulation of miR-138 alone did not affect viability; however it increased the cisplatin sensitivity of four of the eight cell lines by approximately 20% (UMUC9, UMUC14, 253JBV, HT1197, Figure 4B). This is particularly interesting because LNA-anti miR-138 has minimal effect on cell viability by itself (Figure 4A), making cisplatin sensitivity comparisons more robust.

Overexpression of the ABCB1/MDR1 gene coding for the transmembrane transporter P-glycoprotein (P-gp) is a common course of multi drug resistance (MDR). P-gp confers resistance to a broad range of structurally diverse chemotherapeutic drugs by contributing to resistance phenotype by drug efflux from cancer cells [33]. A number of miRNA profiling studies have implicated miRNAs in the MDR phenotype. miR-27a and miR-451 were up-regulated in MDR ovarian cells (A2780DX5) and cervix carcinoma cells (KB-V1). Transfection with antagomirs and mature miR-27a correlated with MDR1/P-gp expression [34]. In esophageal squamous cell carcinoma down-regulation of miR-27a confer sensitivity of both P-gp related (Vincristine (VCR), Adriamycin (ADR)) and non P-gp related drugs (5-Fluorouracil (5-Fu), Cisplatin (CDDP)) [35]. miR-27a is up-regulated in pediatric B-ALL and act as a suppressor of the Tumor suppressor FBW7. FBW7 act as a negative regulator of proliferation by facilitating proteasome degradation of cyclin E, c-Myc, c-Jun and Notch1 [13,36]. Mir-27a down-regulation inhibits proliferation of gastric MKN45 cells *in vitro* and decreases expression of P-gp and confers increased sensitivity against VCR, ADR, 5-Fu and CDDP [25,26]. miR-886 previously proposed to be a vault RNA, a component of the vault complex implicated in cancer drug resistance, was recently shown neither to be a genuine pre-miRNA nor a vault RNA [37]. miR-886 binds directly to PKR (Protein Kinase RNA-activated) and silencing of miR-886 activates PKR and its downstream pathways, eIF2α phosphorylation and the NF-κB pathway, leading to impaired cell proliferation [37]. MicroRNA miR-886-5p inhibits apoptosis of cervical cancer cells (H8, an HPV16-immortalized human cervical squamous epithelial cell line) by down-regulating the production of Bax [38]. miR-193a-3p and miR-492 was up-regulated in bladder cancer cells compared to normal urothelium [39,40].

## Conclusions

In conclusion, we have identified miRs that alter the viability of bladder cancer cells *in vitro*, and that seem to impact on the cisplatin sensitivity *in vitro*. This was shown by both increasing and reducing the miR level in the cells. In clinical samples several miRs were related to Cisplatin response, however may require a larger data set for confirmation. Interestingly several of these seem to impact on molecules relevant for chemo response, however, the network of microRNA interactions is extremely complex and thus requires further study to reveal alterations of importance for the clinical management of patients.

## Competing interests

The authors declare that they have no competing interests.

## Authors’ contributions

IN designed experiments, performed experiments, interpreted results and drafted manuscript. LD conducted statistical analysis and made critical revision to manuscript. MA provided tumor samples and made critical revision to manuscript. KBD, DT, TFØ, MSO, AH and MB designed experiments, interpreted results and made critical revision to manuscript. All authors have read and approved the final manuscript.

## Pre-publication history

The pre-publication history for this paper can be accessed here:

http://www.biomedcentral.com/1755-8794/5/40/prepub

## Supplementary Material

Additional file 1 **Table S1.** Response cohort - patient characteristics and treatment regimens. Click here for file

Additional file 2 **Table S2.** Survival cohort - patient characteristics and treatment regimens. Click here for file

Additional file 3 **Table S3.** miRNA normalization. Normalization of miRNA expression is challenging and therefore we chose to make 4 different normalizations prior to analysis of differentially expressed miRNAs (PD vs.CR) and compare the results. Normalizes used were mammalian U6 (MammU6) and RNU48 RNAs that is often used as normalizes, miR-193b selected using the Norm Finder program, and finally quintile normalization using the qpcrNorm program (Mar J et al. Data-driven Normalization Strategies for qPCR Data. Technical Report, 2008). The results show a large degree of agreement between normalizations. We argue that the Norm Finder approach is the best normalize approach for our data because quintile normalization is better suited for large gene sets and miRNA-193b is more stable than MammU6) and RNU48 across the samples (data not shown). Click here for file

Additional file 4 **Table S4.** Technical validation of miRNA profiling. As a technical evaluation of the Taqman Human Array MicroRNA Cards (LDA analysis) the 6 top ranked miRNAs from the Array analysis were determined using real-time q-RT-PCR (n = 3) (singleplex) and compared to the MicroRNA card analysis. miR-193b expression was used as normalize. Click here for file

Additional file 5 **Table S5.** miRNA normalization. Tree different normalizations prior to analysis of differentially expressed miRNAs (SS vs.LS) were performed to inspect the robustness of the analysis. Normalizes used were mammalian U6 (MammU6), miR-324-3p selected using the Norm Finder program and miR-193b. The results show a large degree of agreement between normalizations. Click here for file

Additional file 6 **Figure S1.** miRNA down-regulation in bladder cell lines. LNA based antagonism were used to silence miR-27a and miR-138 in the bladder cell lines 253JBV and CLR2169. LNA knockdown molecules were reverse Transfected (n = 3) and after 48 h incubation culture media, with or without cisplatin (GI50), was added to the cells (n = 4). Expression of mature miR-27a and miR-138 was determined using real-time Q-PCR (n = 3). LF2000 designate controls using the transfection reagent alone. The 2^-ΔΔCT^ method was used for relative quantification with miR-193b expression normalize. Click here for file
