# Supplementary material for: miRNAs associated with chemo-sensitivity in cell lines and in advanced bladder cancer
Source: BMC Med Genomics. 2012 Sep 6;5:40. doi: 10.1186/1755-8794-5-40 (PMC3473298; doi:10.1186/1755-8794-5-40)
Supplement: Additional file 1 — Table S1. Response cohort - patient characteristics and treatment regimens. [file 1755-8794-5-40-S1.pdf]

Additional file 1: Table S1

Age	Gender	Treatment	RECIST	OS(months)
58	M	g/c II 7014	PD	3,37
62	F	g/c II 7018	PD	3.47
45	M	MVAC up	PD	1
62	M	MVAC up	PD	13,.3
62	F	MVAC up	PD	7.03
54	M	Cis/Carbo/Mtx	PD	0.13
61	M	Cis/Mitox/Mtx	PD	4.63
38	F	cis/pacl/gem 408	CR	22.6
60	M	g/c II 7016	CR	25.53
55	M	g/c II 7019	CR	13.33
49	M	MVAC up	CR	92.13
52	M	MVAC up	CR	49.9
62	F	MVAC up	CR	83.13
69	M	MVAC III 1803	CR	25.1
65	M	Cis/Mitox/Mtx	CR	6.83

MVAC up	Methotrexate 30 mg/m^2, vinblastine 3 mg/m^2, doxorubicin 30 mg/m^2, cisplatin 70 mg/m
MVAC III 1803	Methotrexate 30 mg/m^2, vinblastine 3 mg/m^2, doxorubicin 30 mg/m^2, cisplatin 70 mg/m^2
Cis/Mitox/Mtx	Cisplatin 100 mg/m^2, methotrexate 30 mg/m^2, mitoxantrone 10 mg/m^2
cis/pacl/gem 408	Gemcitabine 1000 mg/m^2, cisplatin 70 mg/m^2, paclitaxel 175 mg/m^2
Cis/Carbo/Mtx	Cisplatin 100 mg/m^2, carboplatin 200 mg/m^2, methotrexate 250 mg/m^2
g/c II	Gemcitabine 1000 mg/m^2, cisplatin 35 mg/m^2
