# Supplementary material for: miRNAs associated with chemo-sensitivity in cell lines and in advanced bladder cancer
Source: BMC Med Genomics. 2012 Sep 6;5:40. doi: 10.1186/1755-8794-5-40 (PMC3473298; doi:10.1186/1755-8794-5-40)
Supplement: Additional file 2 — Table S2. Survival cohort - patient characteristics and treatment regimens. [file 1755-8794-5-40-S2.pdf]

Additional file 2: Table S2

Age	Gender	Treatment	RECIST	OS(months)	Group
54	M	Cis/Carbo/Mtx	PD	0.13	SS
45	M	MVAC up	PD	1	SS
58	M	g/c II 7014	PD	3.37	SS
62	F	g/c II 7018	PD	3.47	SS
71	M	g/c up	PR	4.43	SS
61	M	Cis/Mitox/Mtx	PD	4.63	SS
68	M	g/c III 1826	NE	4.87	SS
57	F	g/c up	NE	5.2	SS
37	M	cis/pacl/gem 410	PR	5.3	SS
65	M	Cis/Mitox/Mtx	CR	6.83	SS
39	F	cis/pacl/gem 402	PD	6.93	SS
62	F	MVAC up	PD	7.03	SS
75	M	MVAC III 1833	NC	7.47	SS
67	M	g/c up	NC	8.4	SS
69	M	g/c III 1805	NC	10.9	SS
62	M	MVAC up	PD	13.23	LS
55	M	g/c II 7019	CR	13.33	LS
38	F	cis/pacl/gem 408	CR	22.6	LS
69	M	MVAC III 1803	CR	25.1	LS
60	M	g/c II 7016	CR	25.53	LS
59	F	cis/pacl/gem 409	PR	49.83	LS
52	M	MVAC up	CR	49.9	LS
74	F	g/c up	PR	51.33	LS
64	M	cis/pacl/gem 406	PR	68.13	LS
62	F	MVAC up	CR	83.13	LS
48	F	MVAC up	NE	87.5	LS
62	M	MVAC up	NE	92	LS
49	M	MVAC up	CR	92.13	LS
65	M	g/c III 1842	PR	97	LS
65	M	g/c II 7015	PR	125.07	LS

MVAC up	Methotrexate 30 mg/m^2, vinblastine 3 mg/m^2, doxorubicin 30 mg/m^2, cisplatin 70 mg/m
MVAC III 1803	Methotrexate 30 mg/m^2, vinblastine 3 mg/m^2, doxorubicin 30 mg/m^2, cisplatin 70 mg/m^2
Cis/Mitox/Mtx	Cisplatin 100 mg/m^2, methotrexate 30 mg/m^2, mitoxantrone 10 mg/m^2
cis/pacl/gem 408	Gemcitabine 1000 mg/m^2, cisplatin 70 mg/m^2, paclitaxel 175 mg/m^2
Cis/Carbo/Mtx	Cisplatin 100 mg/m^2, carboplatin 200 mg/m^2, methotrexate 250 mg/m^2
g/c II	Gemcitabine 1000 mg/m^2, cisplatin 35 mg/m^2
