# Supplementary material for: miRNAs associated with chemo-sensitivity in cell lines and in advanced bladder cancer
Source: BMC Med Genomics. 2012 Sep 6;5:40. doi: 10.1186/1755-8794-5-40 (PMC3473298; doi:10.1186/1755-8794-5-40)
Supplement: Additional file 3 — Table S3. miRNA normalization. Normalization of miRNA expression is challenging and therefore we chose to make 4 different normalizations prior to analysis of differentially expressed miRNAs (PD vs.CR) and compare the results. Normalizes used were mammalian U6 (MammU6) and RNU48 RNAs that is often used as normalizes, miR-193b selected using the Norm Finder program, and finally quintile normalization using the qpcrNorm program (Mar J et al. Data-driven Normalization Strategies for qPCR Data. Technical Report, 2008). The results show a large degree of agreement between normalizations. We argue that the Norm Finder approach is the best normalize approach for our data because quintile normalization is better suited for large gene sets and miRNA-193b is more stable than MammU6) and RNU48 across the samples (data not shown). [file 1755-8794-5-40-S3.pdf]

Additional file 3: Table S3

Normalized to Mamm6U			Normalized to RNU48			Normalized to miR-193b			Quantile normalization		
Detector	Fold Change	Ttest(PD,CR)	Detector	Fold Change	Ttest(PD,CR)	Detector	Fold Change	Ttest(PD,CR)	Detector	Fold Change	Ttest(PD,CR)
hsa-miR-642-4380995	-5,5	0,000	hsa-miR-642-4380995	-5,0	0,001	hsa-miR-642-4380995	-3,8	0,001	hsa-miR-196b-4395326	2,1	0,002
hsa-miR-27a-4373287	-3,5	0,000	hsa-miR-296-5p-4373066	-4,0	0,003	hsa-miR-492-4373217	-2,9	0,003	hsa-miR-186-4395396	1,4	0,002
hsa-miR-27b-4373068	-2,7	0,001	hsa-miR-27a-4373287	-3,0	0,004	hsa-miR-27a-4373287	-1,8	0,003	hsa-miR-138-4395395	3,7	0,003
hsa-miR-24-4373072	-2,9	0,001	hsa-miR-24-4373072	-2,4	0,005	hsa-miR-296-5p-4373066	-2,8	0,003	hsa-miR-639-4380987	-1,7	0,003
hsa-miR-335-4373045	-3,4	0,001	hsa-miR-365-4373194	-2,6	0,006	hsa-miR-944-4395300	-3,6	0,006	hsa-miR-296-5p-4373066	-2,4	0,007
hsa-miR-296-5p-4373066	-4,5	0,001	hsa-miR-335-4373045	-2,9	0,007	hsa-miR-26b*-4395555	-2,6	0,007	hsa-miR-642-4380995	-2,8	0,008
hsa-miR-27b*-4395285	-2,3	0,002	hsa-miR-339-5p-4395368	-2,7	0,007	hsa-miR-193a-5p-4395392	-2,6	0,009	hsa-miR-19b-1*-4395536	0,8	0,008
hsa-miR-576-3p-4395462	-3,3	0,002	hsa-miR-625-4395542	-2,3	0,007	hsa-miR-886-3p-4395305	-2,1	0,020	hsa-miR-944-4395300	-3,7	0,009
hsa-miR-218-4373081	-3,1	0,002	hsa-miR-193a-5p-4395392	-3,8	0,008	hsa-miR-576-3p-4395462	-1,6	0,020	hsa-miR-331-5p-4395344	1,2	0,012
hsa-miR-324-5p-4373052	-2,6	0,003	hsa-miR-199a-5p-4373272	-2,6	0,010	hsa-miR-923-4395264	-2,5	0,021	hsa-miR-576-3p-4395462	-0,9	0,012
hsa-miR-365-4373194	-3,1	0,003	hsa-miR-576-3p-4395462	-2,8	0,010	hsa-miR-186-4395396	1,0	0,022	hsa-miR-339-3p-4395295	1,5	0,013
hsa-miR-625-4395542	-2,8	0,003	hsa-miR-124-4373295	-3,2	0,010	hsa-miR-625-4395542	-1,2	0,022	hsa-miR-628-5p-4395544	1,4	0,015
hsa-miR-183*-4395381	-2,4	0,004	hsa-miR-27b-4373068	-2,2	0,011	hsa-miR-24-4373072	-1,3	0,022	hsa-miR-492-4373217	-1,8	0,016
hsa-miR-34a-4395168	-2,6	0,004	hsa-miR-492-4373217	-3,9	0,011	hsa-miR-639-4380987	-1,8	0,023	hsa-miR-24-4373072	-0,9	0,018
hsa-miR-181a*-4373086	-3,5	0,005	hsa-miR-218-4373081	-2,6	0,011	hsa-miR-199a-5p-4373272	-1,2	0,037	hsa-miR-193a-5p-4395392	-1,8	0,023
hsa-miR-492-4373217	-4,1	0,005	hsa-miR-330-3p-4373047	-1,9	0,013	hsa-miR-138-4395395	2,5	0,037	hsa-miR-335-4373045	-1,0	0,028
hsa-miR-374b-4381045	-2,4	0,005	hsa-miR-944-4395300	-3,7	0,014	hsa-miR-124-4373295	-1,8	0,040	hsa-miR-27a-4373287	-1,3	0,036
hsa-miR-193a-5p-4395392	-4,3	0,006	hsa-miR-34a-4395168	-2,1	0,016	hsa-miR-218-4373081	-1,5	0,042	hsa-miR-345-4395297	1,6	0,039
hsa-miR-330-3p-4373047	-2,4	0,006	hsa-miR-324-5p-4373052	-2,1	0,019	hsa-miR-152-4395170	-1,2	0,043	hsa-miR-923-4395264	-1,6	0,044
hsa-miR-20a*-4395548	-3,2	0,006	hsa-miR-923-4395264	-2,9	0,022	hsa-miR-339-3p-4395295	0,9	0,051	hsa-miR-432-4373280	1,7	0,044
hsa-miR-136*-4395211	-3,0	0,006	hsa-miR-192-4373108	-2,4	0,022				hsa-miR-574-3p-4395460	1,4	0,047
hsa-miR-639-4380987	-3,9	0,006	hsa-miR-639-4380987	-2,3	0,024				hsa-miR-191-4395410	0,9	0,053
hsa-miR-199a-5p-4373272	-3,1	0,006	hsa-miR-886-3p-4395305	-3,3	0,024						
hsa-miR-923-4395264	-4,5	0,007	hsa-miR-422a-4395408	-1,9	0,025						
hsa-miR-944-4395300	-5,3	0,007	hsa-miR-206-4373092	-1,9	0,055						
