# Supplementary material for: miRNAs associated with chemo-sensitivity in cell lines and in advanced bladder cancer
Source: BMC Med Genomics. 2012 Sep 6;5:40. doi: 10.1186/1755-8794-5-40 (PMC3473298; doi:10.1186/1755-8794-5-40)
Supplement: Additional file 4 — Table S4. Technical validation of miRNA profiling. As a technical evaluation of the Taqman Human Array MicroRNA Cards (LDA analysis) the 6 top ranked miRNAs from the Array analysis were determined using real-time q-RT-PCR (n = 3) (singleplex) and compared to the MicroRNA card analysis. miR-193b expression was used as normalize. [file 1755-8794-5-40-S4.pdf]

Additional file 4: Table S4

Target miR	LDA Analysis Log2 (PD vs. CR)	QPCR singelplex Log2 (PD vs. CR)
Hsa-miR-27a	-1.8	-1.9
Hsa-miR-193a-5p	-2.6	-2.8
Hsa-miR-296-5p	-2.8	-2.8
Hsa-miR-492	-2.9	-2.7
Hsa-miR-642	-3.8	-3.9
Hsa-miR-944	-3.6	-3.2
