# Supplementary material for: miRNAs associated with chemo-sensitivity in cell lines and in advanced bladder cancer
Source: BMC Med Genomics. 2012 Sep 6;5:40. doi: 10.1186/1755-8794-5-40 (PMC3473298; doi:10.1186/1755-8794-5-40)
Supplement: Additional file 5 — Table S5. miRNA normalization. Tree different normalizations prior to analysis of differentially expressed miRNAs (SS vs.LS) were performed to inspect the robustness of the analysis. Normalizes used were mammalian U6 (MammU6), miR-324-3p selected using the Norm Finder program and miR-193b. The results show a large degree of agreement between normalizations. [file 1755-8794-5-40-S5.pdf]

Additional file 5: Table S5

Normalized to hsa-miR-324-3p-4395272			Normalized to MammU6-4395470			Normalized to hsa-miR-193b-4395478		
Detector	Fold Change	Ttest	Detector	Fold Change	Ttest	Detector	Fold Change	Ttest
hsa-miR-410-4378093	-1,15	0,011	hsa-miR-886-5p-4395304	-2,22	0,005	hsa-miR-886-5p-4395304	-1,64	0,007
hsa-miR-886-3p-4395305	-1,60	0,011	hsa-miR-27a*-4395556	-1,78	0,008	hsa-miR-944-4395300	-1,80	0,018
hsa-miR-944-4395300	-1,80	0,018	hsa-miR-410-4378093	-1,90	0,014	hsa-miR-923-4395264	-1,50	0,018
hsa-miR-923-4395264	-1,50	0,018	hsa-miR-380*-4373021	-1,97	0,015	hsa-miR-410-4378093	-1,32	0,041
hsa-miR-196b-4395326	1,24	0,019	hsa-miR-886-3p-4395305	-2,35	0,024	hsa-miR-886-3p-4395305	-1,78	0,051
hsa-miR-138-4395395	1,94	0,022	hsa-miR-222-4395387	-1,45	0,033	hsa-miR-138-4395395	1,77	0,052
hsa-miR-345-4395297	0,91	0,055	hsa-miR-923-4395264	-2,32	0,036	hsa-miR-222-4395387	-0,87	0,069
hsa-miR-886-5p-4395304	-1,47	0,058	hsa-miR-21*-4395549	-1,22	0,046	hsa-miR-223-4395406	-1,35	0,103
hsa-miR-186-4395396	0,81	0,071	RNU44-4373384	-1,11	0,048	hsa-miR-146b-5p-4373178	-1,08	0,113
