# Supplementary figures and images for: miRNAs associated with chemo-sensitivity in cell lines and in advanced bladder cancer
Source: BMC Med Genomics. 2012 Sep 6;5:40. doi: 10.1186/1755-8794-5-40 (PMC3473298; doi:10.1186/1755-8794-5-40)

Additional file 6: Figure S1

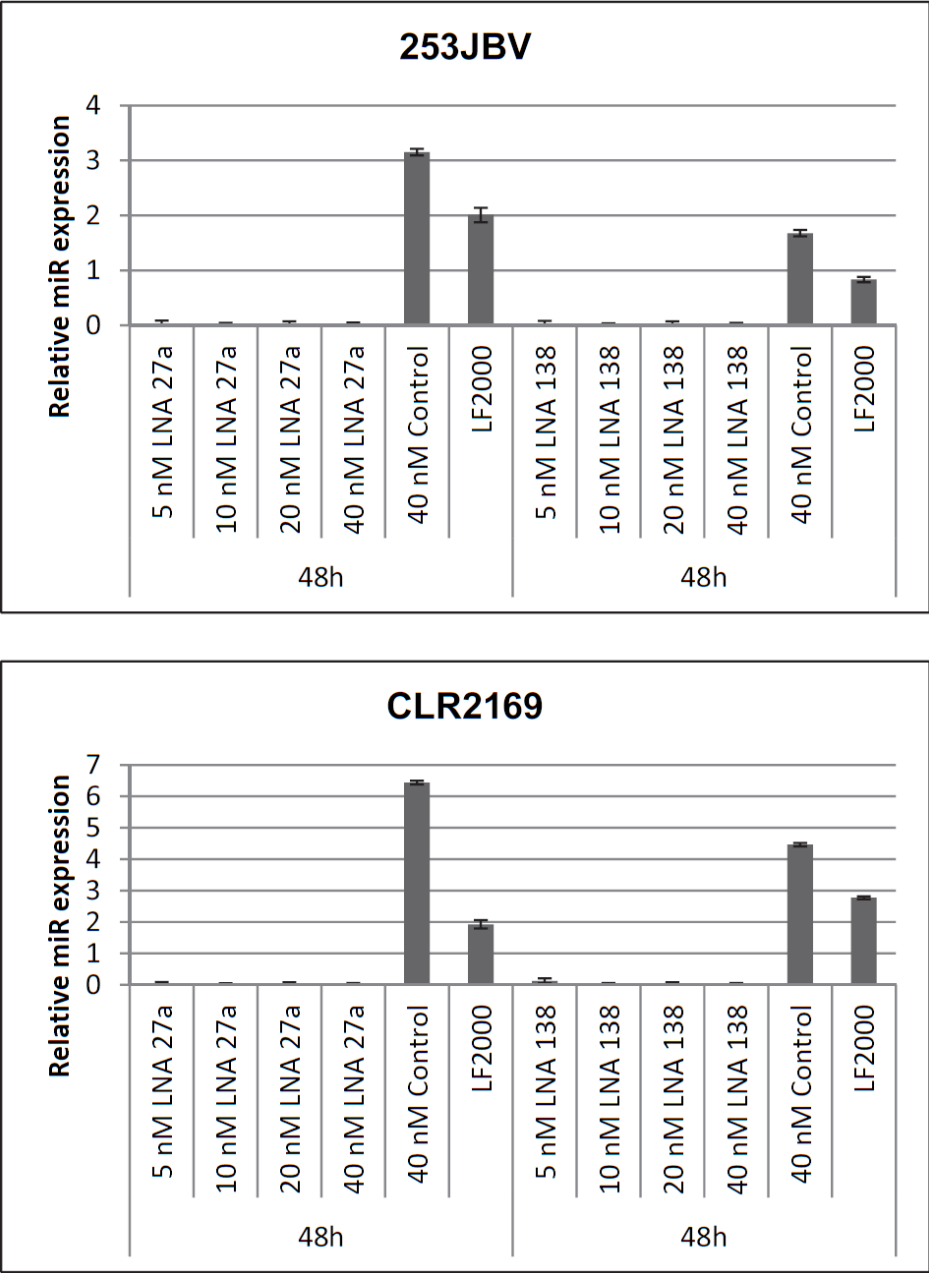

Supplement: Additional file 6 — Figure S1. miRNA down-regulation in bladder cell lines. LNA based antagonism were used to silence miR-27a and miR-138 in the bladder cell lines 253JBV and CLR2169. LNA knockdown molecules were reverse Transfected (n = 3) and after 48 h incubation culture media, with or without cisplatin (GI50), was added to the cells (n = 4). Expression of mature miR-27a and miR-138 was determined using real-time Q-PCR (n = 3). LF2000 designate controls using the transfection reagent alone. The 2-ΔΔCT method was used for relative quantification with miR-193b expression normalize. [file 1755-8794-5-40-S6.pdf]
